# Functions of IFNλs in Anti-Bacterial Immunity at Mucosal Barriers

**DOI:** 10.3389/fimmu.2022.857639

**Published:** 2022-05-18

**Authors:** Noémie Alphonse, Ruth E. Dickenson, Abrar Alrehaili, Charlotte Odendall

**Affiliations:** ^1^Department of Infectious Diseases, School of Immunology and Microbial Sciences, King’s College London, London, United Kingdom; ^2^Immunoregulation Laboratory, Francis Crick Institute, London, United Kingdom

**Keywords:** type III interferon, IFNλs, interferon signaling, epithelial barrier, bacterial infection, mucosal barrier

## Abstract

Type III interferons (IFNs), or IFNλs, are cytokines produced in response to microbial ligands. They signal through the IFNλ receptor complex (IFNLR), which is located on epithelial cells and select immune cells at barrier sites. As well as being induced during bacterial or viral infection, type III IFNs are produced in response to the microbiota in the lung and intestinal epithelium where they cultivate a resting antiviral state. While the multiple anti-viral activities of IFNλs have been extensively studied, their roles in immunity against bacteria are only recently emerging. Type III IFNs increase epithelial barrier integrity and protect from infection in the intestine but were shown to increase susceptibility to bacterial superinfections in the respiratory tract. Therefore, the effects of IFNλ can be beneficial or detrimental to the host during bacterial infections, depending on timing and biological contexts. This duality will affect the potential benefits of IFNλs as therapeutic agents. In this review, we summarize the current knowledge on IFNλ induction and signaling, as well as their roles at different barrier sites in the context of anti-bacterial immunity.

## 1 Type III Interferon Induction and Signaling

### 1.1 Type III Interferons and Their Receptor

Type III interferons (IFNs), also called IFNλs, are the most recent addition to the IFN family. These class II cytokines include two more members: type I and II IFNs. Type I IFNs include IFNαs and IFNβ and are functionally similar to IFNλs. They are produced by many cell types and signal through the IFNα Receptor (IFNAR), which is expressed on nearly every nucleated cell (1, 2). Type II IFN only includes IFNγ and is the most phylogenetically and functionally distinct (3, 4). The type III IFN family is comprised of 4 members in humans: IFNλ1 (IL-29), IFNλ2 (IL-28A), IFNλ3 (IL-28B), and the most recently identified IFNλ4. Mice express IFNλ2 and IFNλ3, but murine IFNλ1 and IFNλ4 are pseudogenes (5). Type III IFNs are induced by both epithelial and immune cells (6, 7), in particular dentritic cells (DC) (8–11). Type I and III IFNs are produced in response to the detection of Microbe-Associated Molecular Patterns (MAMPs) by host Pattern Recognition Receptors (PRRs) (12–17). Secreted IFNλs signal in an autocrine and paracrine manner through a common heterodimeric receptor, IFNLR, composed of the high affinity type III IFN receptor (IFNLR1, also known as IL-28Ra) and the Interleukin 10 Receptor 2 (IL-10R2) (18). IFNLR is mostly expressed at barrier sites and was initially thought to be only present on epithelial cells (19–21). However, increasing evidence has demonstrated the expression of IFNLR on multiple immune cell subsets such as neutrophils (22), macrophages (23), plasmacytoid dentritic cells (pDCs) (8, 24) and lymphocytes (5, 25, 26). Human natural killer (NK) cells do not appear to express IFNLR (27), whereas mouse NK cells express IFNLR1 and potently respond to IFNλ (28). This selective distribution of IFNLR distinguishes type III IFNs from the ubiquitously sensed type I IFNs (1, 29).

### 1.2 Production of Type III IFNs in Response to Bacterial Ligands

MAMPs are conserved microbe-specific structures that are produced by both pathogenic and non-pathogenic microorganisms. They include nucleic acids and bacterial cell components like flagellin, present in the flagella of motile bacteria (30); peptidoglycan, found in the cell wall of most bacteria (31); or lipopolysaccharide (LPS) from the outer membrane of Gram-negative bacteria (32). Detection of both Gram-positive and Gram-negative bacteria by epithelial and innate immune cells was shown to induce type III IFNs to similar or greater levels than type I IFNs (13, 16, 33–36). IFNλs are induced *via* stimulation of all PRRs that induce type I IFN (37), including the cytosolic DNA and RNA sensors cGAS (38–43) RIG-I-like receptors (RLRs) (44) and endosomal Toll-like receptors (TLRs) (16, 35, 45). However, several signaling pathways have been shown to preferentially drive IFNλ expression (16, 33, 35, 46). For example, following detection of LPS by TLR4, IFNβ and IFNλ are induced *via* distinct mechanisms. While the induction of IFNβ does not occur until TLR4 reaches endosomes, and is independent of the adaptor MyD88 (47–49), IFNλ production occurs from the plasma membrane and requires MyD88 (16). Similarly, TLR2 and TLR5 that detect bacterial components from the plasma membrane do not strongly induce type I IFNs (16, 50–52), but potently induce type III IFN expression (16). Therefore, IFNλs seem to be preferentially induced in response to ligands of bacterial origin, hinting at possible roles of these IFNs in antibacterial immunity.

### 1.3 IFNλ-Mediated Signaling and Anti-Bacterial ISGs

Although type I and type III IFNs bind unique receptors, the signaling pathways downstream of IFNLR and IFNAR activation are similar. In both cases, phosphorylated Janus kinase (JAK) family proteins activate Signal Transducer and Activator of Transcription 1 (STAT1) and STAT2. Activated STAT1/2 form a complex with the IFN regulatory factor 9 (IRF9), called the IFN stimulated gene factor 3 (ISGF3). ISGF3 translocates into the nucleus where it binds IFN-stimulated response elements (ISREs) located in the promoters of IFN-stimulated genes (ISGs) (19, 29). Despite these similarities, the combination of JAK kinases activated by type I and III IFNs may be different. Some studies have suggested that JAK2 may mediate IFNLR, but not IFNAR, signaling (22, 35, 53). In addition, Tyrosine kinase 2 (TYK2) is required for type I IFN signaling, but seems to be dispensable for IFNλ signaling (54–56).

Hundreds of ISGs are expressed in response to IFNs and can modulate innate and adaptive immunity to promote microbial clearance. The ISGs expressed in response to type I and type III IFNs overlap greatly, however the kinetics of their production differ. Type I IFNs typically induce a strong, rapid expression of ISGs, whereas the type III IFN response is slower and of a lesser magnitude (19, 29, 57–61). Although the functions of IFNλ and ISGs have been predominantly studied in the context of viral infection (62, 63), emerging evidence indicates a protective role of some ISGs in bacterial infection. For example, the anti-viral IFN induced transmembrane (IFITM) proteins have been shown to restrict *Mycobacterium tuberculosis* intracellular growth (64) and the ISG Viperin inhibits the entry of *Shigella* into epithelial cells (65). Additionally, guanylate-binding proteins (GBPs) bind the LPS of Gram-negative bacteria, facilitating activation of the non-canonical inflammasome (66–69). GBPs also interfere with actin-based motility of *Shigella flexneri*, hindering bacterial dissemination (70, 71). Given their emerging antibacterial roles, ISGs are attractive targets for bacterial virulence factors (72). This is exemplified by the *Shigella* ubiquitin ligase IpaH9.8, which targets GBPs for proteosomal degradation (69, 71, 73).

The production of IFNλs and ISGs in response to bacterial ligands, in conjunction with the privileged localisation of IFNLR, raises interest regarding the function of IFNλ at barrier sites. Here, we summarize the roles of IFNλ in mucosal epithelia during disease and homeostasis.

## 2 Type III IFNs in Anti-Bacterial Mucosal Immunity

### 2.1 Type III IFNs and Epithelial Barriers

#### 2.1.1 Intestinal Epithelial Barriers

Compartmentalisation by epithelial barriers is critical in the intestine, as they not only protect the host from potential pathogens but also separate the underlying tissue from foreign material ingested by the host. IFNλs protect intestinal epithelial barrier integrity (16, 74, 75). In a mouse model of colitis induced by dextran sulfate sodium (DSS), IFNλs were shown to control the proliferation of intestinal epithelial cells and accelerate intestinal mucosal healing, which reduced epithelial cell damage (15, 22, 76).

**Figure 1:**
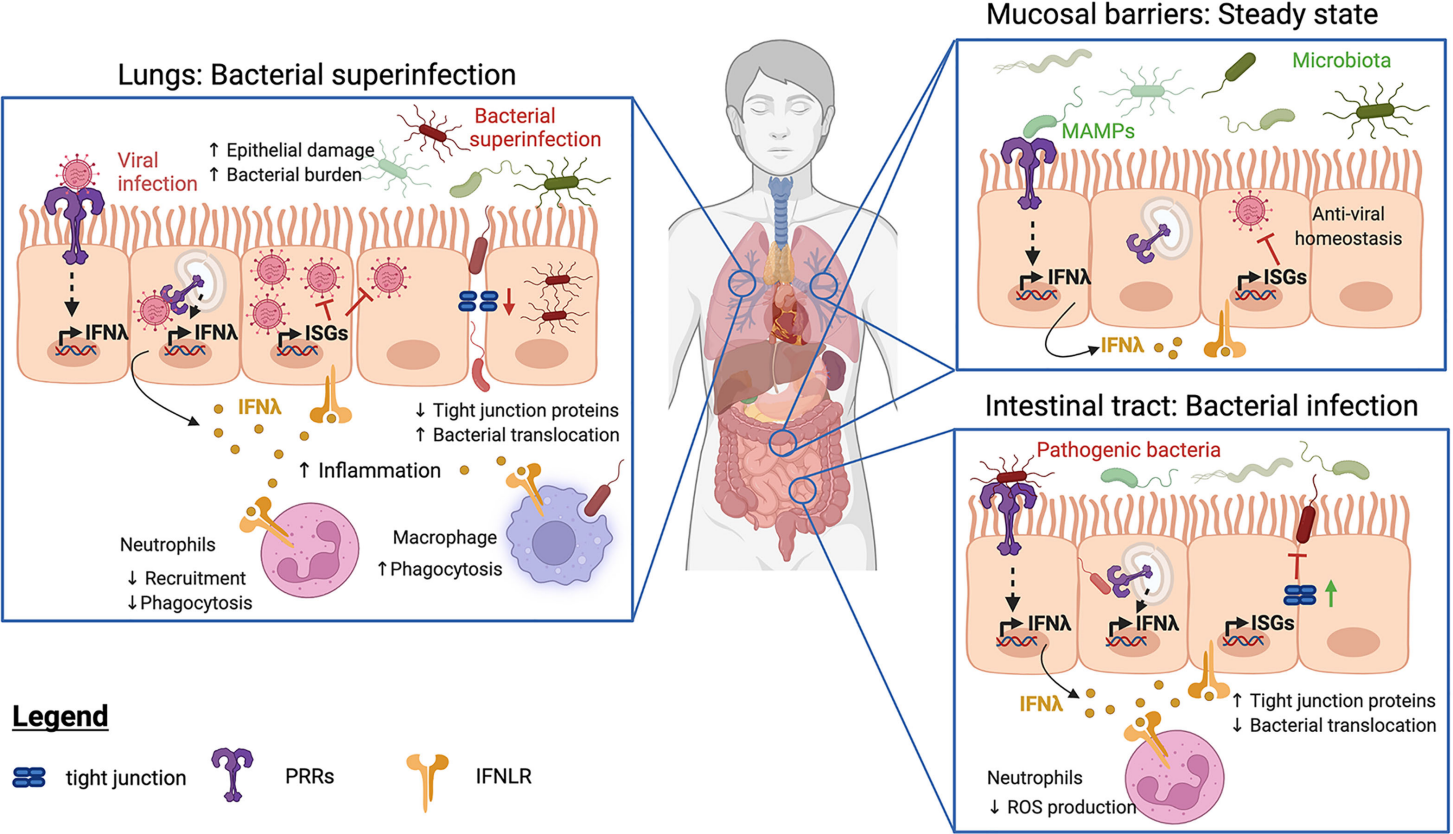
Functions of IFNλ at barrier sites at steady state and during bacterial infection. At barrier sites, detection of Microbe-Associated Molecular Patterns (MAMPs) by Pattern Recognition Receptors (PRRs) triggers IFNλ production (in yellow) and subsequent expression of IFN-stimulated genes (ISGs). IFNλ can enhance (green lines) or inhibit (red lines) host immune responses. In infected or inflamed lungs (left panel), IFNλ and ISGs restrict neutrophil recruitment, resulting in increased bacterial burdens. IFNλ also decreases tight junction protein production, facilitating bacterial translocation and promoting bacterial superinfections. In contrast, IFNλ promotes the phagocytic activity of macrophages. During bacterial infections of the intestinal tract (lower right panel), IFNλ strengthens tight junction proteins, preventing bacterial transmigration. IFNλ also impairs neutrophil recruitment and reactive oxygen species (ROS) production to limit tissue destruction. At both sites (top right panel), steady-state detection of the bacterial microbiota induces the production of IFNλs, whose signaling drives homeostatic expression of ISGs. The resulting basal ISG response protects against viral infections.

The protective role of IFNλ in intestinal epithelia extends to the context of bacterial infection. Treatment with IFNλ1 was shown to increase trans-epithelial electrical resistance (TEER) in an *in vitro* model of barrier integrity using polarized T84 colonic epithelial cells (16). Invasive enteric pathogens such as *S. flexneri* and *Salmonella enterica* serovar Typhimurium disrupt epithelial barriers to aid bacterial dissemination. While TEER dropped upon infection with these bacteria in control conditions, it was maintained following IFNλ1 pre-treatment. In addition, IFNλ treatment prevented *S. flexneri* and *S.* Typhimurium transmigration, showing that IFNλ protects intestinal epithelial barriers from infection and damage mediated by invasive bacteria *in vitro* (16) (**Figure 1**). The molecular mechanism of action of IFNλs-mediated protection of barriers has not been uncovered, but intracellular tight junction proteins like claudin-1 were shown to be upregulated by IFNλ2 (75). Whether these observations in cell culture extend to *in vivo* models of bacterial infection remains to be determined.

#### 2.1.2 Airways Epithelial Barriers

While type III IFNs appear to protect against enteric bacterial infections, their roles in the airway epithelium are unclear. At steady state, IFNλs modulate immune cell responses to decrease inflammation (24, 25, 77, 78). However, during infection, the activities of type III IFNs on lung epithelial cells were also shown to increase inflammation and compromise barrier function (79–82) (**Figure 1**). During infection with *Klebsiella pneumoniae*, IFNλ treatment lowered epithelial barrier integrity *in vitro*, facilitating neutrophil transmigration and bacterial translocation. Moreover, *Ifnlr1^-/-^* mice were protected from *Klebsiella*-induced pneumonia (79). Similarly, IFNλ exacerbated lung inflammatory pathology in a mouse model of *Bordetella pertussis* infection (80). In this study, antibody neutralization of IFNλ, as well as deletion of IFNLR, led to reduced lung inflammatory pathology (80), congruent with a detrimental role of type III IFN in bacteria-infected lungs.

Compromised barrier functions and increased inflammation during viral infection in the lungs can result in complications such as bacterial superinfection (81–84). Infection with Influenza A virus (IAV) or intratracheal instillation of poly (I:C), a synthetic ligand that mimics viral dsRNA, were shown to up-regulate IFNλ2 and IFNλ3. This led to compromised barrier function and promoted superinfection by *Staphylococcus aureus* (81) and *S. pneumoniae* (82). Greater barrier damage and higher bacterial burdens were found in mice treated with poly (I:C), a phenotype reversed in mice lacking IFNLR. Moreover, the co-administration of IFNλ and R848 (a TLR7 agonist that mimics viral ssRNA), but not IFNλ alone, increased sensitivity to *S. aureus* infection. This suggests that IFNλ promotes superinfections in inflamed lungs by driving inflammation and compromising barrier function (81) (**Figure 1**).

IFNλ was also shown to impair neutrophil recruitment and phagocytosis during recovery from IAV infection (85). This enhanced superinfections with methicillin-resistant *S. aureus* (MRSA) or *S. pneumoniae* (82, 83). Consequently, mice lacking IFNLR that were infected with IAV were less susceptible to superinfections (82, 83).

Although type III IFNs can be protective in the intestinal epithelium and healthy lung, it is evident that they can have a detrimental role in inflamed airways. These findings emphasize that biological context is crucial, and further research is required to understand the nuanced effects of IFNs at epithelial barriers.

### 2.2 Type III IFNs and the Microbiota

Mucosal barriers serve to protect the host in a multitude of ways. As well as forming a physical barrier, they maintain a tolerance to the commensal microorganisms that make up the microbiota. This population benefits the host by inhibiting infection by pathogenic species, as well as contributing to host metabolism (86).

#### 2.2.1 Intestinal Microbiota

At steady-state, ISGs are detected in mouse and human intestinal epithelial cells (87–90). The expression of ISGs is dependent on PRR-mediated sensing of the microbiota, particularly in the ileum, and expression of specific ISGs was found to be ablated following antibiotic treatment to eliminate the microbiota (87, 90). Interestingly, homeostatic ISG expression was shown to control enteric viral infections (89), which benefits the host (**Figure 1**).

#### 2.2.2 Airway Microbiota

Although the pulmonary microbiota is poorly understood (91–93), a homeostatic ISG response has also been found in mouse lungs (89, 94). Emerging evidence demonstrates that IFNλ is also important for regulating the microbiota and influencing infection at this mucosal barrier in humans (**Figure 1**).

One study investigated the microbiota composition using random sampling of bronchoalveolar lavage fluid (BALF) from lung transplant recipients. Post-transplant lung microbiota were categorised into four ‘pneumotypes’. In balanced pneumotype, BALF samples analysis of differential expression of host genes, revealed that *Ifnlr1* was upregulated, relative to the other groups. This balanced pneumotype was associated with the lowest risk of infection and allogenic responses, resulting in a lower clinical risk (95). Moreover, in a study assessing the contribution of nasal commensal bacteria in antiviral defense against IAV infection in the upper respiratory tract, the commensal bacterium *Staphylococcus epidermidis* was shown to reduce host susceptibility to IAV infection by inducing the expression of IFNλ (96).

### 2.3 Type III IFNs and Immune Cells

It was initially believed that the IFNLR was solely expressed by epithelial cells, but it has since been demonstrated to be present on immune cells. Neutrophils (22), macrophages (23), pDCs (8, 24), T cells, B cells (5, 25, 26) and mouse NK cells (28) express IFNLR. These cells exhibit limited basal expression of IFNLR, but can rapidly increase expression upon stimulation of TLRs (10, 22, 28), T cell receptor (TCR) (23, 26) or B cell receptor (BCR) (26).

#### 2.3.1 Neutrophils

Neutrophils are phagocytes that can engulf foreign materials and pathogens. They also potently produce microbicidal reactive oxygen species (ROS) and promote inflammation *via* degranulation. However, excessive or prolonged neutrophil activation is a hallmark of many inflammatory pathologies. IFNLR is expressed at higher levels in human and mouse neutrophils than other immune or epithelial cells (22, 25, 78, 97), and its expression in neutrophils is further upregulated by bacterial ligands such as LPS (22). While IFNβ elicits pro-inflammatory activities in neutrophils (78), IFNλs cultivate an anti-inflammatory state by inhibiting neutrophil recruitment (22, 25, 85). Crucially, IFNλ treatment of bone marrow derived neutrophils diminished ROS production and degranulation but did not affect protective cytokine production or phagocytosis. This was protective in the intestine in a mouse model of DSS-induced colitis (22).

Neutrophils are efficient phagocytes and are essential to the resolution of infection. Despite their crucial roles, the functions of IFNλ in neutrophils in the context of bacterial infections have seldom been addressed. In models of bacterial superinfection in murine lungs, IFNλ was shown to reduce the phagocytic abilities of neutrophils which impaired bacterial clearance, exposing the host to infection (85). However, during infection with *Pseudomonas aeruginosa*, an IFNλ2-mediated decrease in neutrophil recruitment was protective and resulted in less epithelial damage (98) (**Figure 1**). As such, the balance between inhibiting neutrophil-mediated inflammatory damage and promoting bacterial clearance should be carefully considered when evaluating the therapeutic potential of IFNλ.

#### 2.3.2 Macrophages

Macrophages are phagocytes that engulf and destroy bacteria. They can also stimulate the adaptive immune system *via* the presentation of foreign antigens to T cells. Exposure to IFNλ increases their phagocytic activity and the production of proinflammatory cytokines and chemokines (23). This promotes bacterial clearance during infection; IFNλ1 was shown to enhance *S. aureus* uptake in macrophages, and increase bacterial killing (99) (**Figure 1**).

Interestingly, when monocytes are incubated with Granulocyte Macrophage Colony-Stimulating Factor (GM-CSF) or Macrophage Colony-Stimulating Factor (M-CSF) to respectively produce M1- or M2-shifted macrophages, the differentiated macrophages respond differently to IFNλ3. GM-CSF differentiated macrophages demonstrated greater IFNLR1 expression and had increased levels of STAT1 phosphorylation and ISG expression in response to IFNλ3 than M-CSF differentiated macrophages (23). Moreover, GM-CSF differentiated macrophages exhibited a pro-inflammatory profile and were more potent at recruiting leukocytes and NK cells upon IFNλ3 stimulation. Interestingly, when stimulated with type I IFN, these macrophages did not exhibit a pro-inflammatory phenotype and were incapable of recruiting leukocyte and NK cells (23).

#### 2.3.3 Other Immune Cells

IFNλ also alters the function of pDCs (8, 24), T and B lymphocytes (5, 26) and mouse NK cells (28). However, whether these effects have an impact on bacterial infections has yet to be explored. pDCs are antigen presenting cells which secrete cytokines that recruit and activate cells of the adaptive immune system. IFNλ3 was shown to prolong the survival of human pDCs *in vitro*, as well as increase their immunoreactivity and ISG response (100). IFNλ was also demonstrated to enhance activation of, and antibody production by, human B lymphocytes (101, 102) and skew the T helper 1 (Th1)/Th2 balance to a Th1-pro-inflammatory response (103–105). Finally, in an *in vivo* model of bacterial-induced inflammation, IFNλ signaling in mouse NK cells was shown to induce IFNγ production, which promoted inflammation. Although enhanced activation of immune cells may promote bacterial clearance, the dangers of prolonged inflammation and tissue destruction must be considered. As such, studying the role of IFNλ in the resolution of inflammatory responses to infection is essential to uncover whether it causes any detrimental effects.

## 3 Concluding Remarks

IFNλs are crucial mediators of inflammation. They are produced by a variety of cell types in response to both commensal and pathogenic microorganisms. Although the IFNLR receptor is found on a restricted subset of cells, the localisation of these cells at barrier sites makes them optimally suited as gatekeepers of immune responses (summarized in **Figure 1**). Homeostatic IFNλ production in response to the microbiota promotes a resting antiviral state and strengthens epithelial barrier integrity in the intestines and the lungs. IFNλ has diverse effects in health and disease, with the outcome depending on the biological context. In the intestine, IFNλ appears to be protective during bacterial infection, inhibiting the invasion of enteric pathogens *in vitro.* In contrast, in the respiratory tract, IFNλ production during viral infection promotes inflammation and can lead to bacterial superinfections. In addition to the lungs and intestinal tract, mucosal epithelial barriers are also present in the genitourinary tract. IFNλ is produced and protective against viral infection in the vaginal and cervical epithelium (106–108). Whether type III IFNs are protective against genitourinary bacterial infections warrants further investigation.

IFNλ also has inverse effects on different immune cells. While it dampens neutrophil recruitment and phagocytosis, it enhances bacterial uptake and killing by macrophages. As they are intrinsically involved in innate and adaptive immune responses, it is important that IFNλs are not studied in isolation. Although IFNλ and the other IFN family members are functionally distinct, they exert antagonistic or synergistic influences on each other (109–111). For example, epithelial cells lacking type III IFN signaling were shown to be more responsive to type I IFN. Conversely, depletion of type I IFN signaling negatively regulates the sensitivity of cells to IFNλ (109). Finally, defining the bidirectional relationship that occurs between the gut microbiota and the lung IFN response will be key to our understanding of anti-bacterial immunity at barrier sites. This review illustrates the need for further research into the functions of IFNλ at barrier sites in the context of bacterial infection and mandates further exploration into this field.

## Author Contributions

All authors contributed to the article and figure and approved the submitted version.

## Funding

NA is supported by a studentship from the King’s College London/Francis Crick Institute partnership which receives its core funding from Cancer Research UK (FC001206), the UK Medical Research Council (FC001206) and the Wellcome Trust (FC001206). RD is supported by a studentship from the UK Medical Research Council (MR/N013700/1). AA is supported by a studentship from the Ministry of Higher Education in the Kingdom of Saudi Arabia. CO is supported by a Sir Henry Dale Fellowship from the Royal Society and the Wellcome Trust (206200/Z/17/Z). For the purpose of open access, the author has applied a CC BY public copyright licence to any Author Accepted Manuscript version arising from this submission.

## Conflict of Interest

The authors declare that the research was conducted in the absence of any commercial or financial relationships that could be construed as a potential conflict of interest.

## Publisher’s Note

All claims expressed in this article are solely those of the authors and do not necessarily represent those of their affiliated organizations, or those of the publisher, the editors and the reviewers. Any product that may be evaluated in this article, or claim that may be made by its manufacturer, is not guaranteed or endorsed by the publisher.
